# Evidence based recommendations for diagnosis and treatment of tumor necrosis factor receptor-1 associated periodic syndrome (TRAPS)

**DOI:** 10.1186/1546-0096-12-S1-P80

**Published:** 2014-09-17

**Authors:** Nienke Ter Haar, Paul Brogan, Gilles Grateau, Jordi Anton-Lopez, Karyl Barron, Luca Cantarini, Joost Frenkel, Caroline Galeotti, Veronique Hentgen, Michael Hofer, Tilmann Kallinich, Isabelle Kone-Paut, Jasmin Kümmerle-Deschner, Huri Ozdogan, Seza Ozen, Anna Simon, Yosef Uziel, Carine Wouters, Brian Feldman, Bas Vastert, Nico Wulffraat, Helen Lachmann, Marco Gattorno

**Affiliations:** 1 Laboratory for Translational Immunology, University Medical Center Utrecht, Utrecht, Netherlands; 2 Department of Rheumatology, UCL Institute of Child Health, London, UK; 3 Centre national de référence des amyloses d'origine inflammatoire et de la fièvre , Hôpital Tenon,AP-HP, université Pierre-et-Marie-Curie, Paris, France; 4 Hospital Sant Joan de Déu, Universitat de Barcelona, Barcelona, Spain; 5 NIH, Bethesda, USA; 6 Policlinico Le Scotte, University of Siena, Siena, Italy; 7 Department of Pediatrics, University Medical Center Utrecht, Utrecht, Netherlands; 8 Reference Centre for Autoinflammatory Disorders CEREMAI, Bicêtre Hospital, University of Paris SUD, Paris, France; 9 Centre Hospitalier de Versailles, Le Chesnay Cedex, France; 10 Department of Pediatrics, University of Lausanne and, University of Geneva, Switzerland; 11 Charite University Medicine, Berlin, Germany; 12 Department of Pediatric Rheumatology, Reference Centre for Autoinflammatory Disorders CEREMAI, Bicêtre Hospital, University of Paris SUD, Paris, France; 13 Klinik für Kinder- und Jugendmedizin, Abteilung für pädiatrische Rheumatologie, Autoinflammation Reference Center Tübingen, Universitätsklinikum Tübingen, Tübingen, Germany; 14 Cerrahpasa Ic Hastaliklari Klinigi, Istanbul, Turkey; 15 Department of Pediatrics, Hacettepe University Faculty of Medicine, Ankara, Turkey; 16 Department of Medicine, Radboudumc, Nijmegen, Netherlands; 17 Department of Pediatrics, Sapir Medical Center, Kfar Saba, Tel-Aviv University, Sackler School of Medicine, Tel-Aviv, Israel; 18 University Hospital Leuven, Leuven, Belgium; 19 Division of Rheumatology, The Hospital for Sick Children, Toronto, Canada; 20 Department of Pediatric Immunology, University Medical Center Utrecht, Utrecht, Netherlands; 21 National Amyloidosis Centre, University College London Medical School, London, UK; 22 G. Gaslini Institute, Genova, Italy

## Introduction

Tumor necrosis factor receptor-1 associated periodic syndrome (TRAPS) is a rare hereditary autoinflammatory syndrome that can lead to significant morbidity. Evidence-based guidelines are lacking and management is mostly based on physician’s experience. Consequently, treatment regimens differ throughout Europe. In 2012, a European initiative called SHARE (*Single Hub and Access point for pediatric Rheumatology in Europe*) was launched to optimize and disseminate diagnostic and management regimens in Europe for children and young adults with rheumatic diseases.

## Objectives

One of the aims of SHARE was to provide evidence based recommendations for diagnosis and treatment of TRAPS.

## Methods

Evidence based recommendations were developed using the European League Against Rheumatism (EULAR) standard operating procedure. An expert committee was instituted, consisting of pediatric and adult rheumatologists. The expert committee defined search terms for the systematic literature review. Two independent experts scored articles for validity and level of evidence. Recommendations derived from the literature were evaluated by an online survey. Those with less than 80% agreement on the online survey or with relevant comments of the experts were reformulated. Subsequently, all recommendations were discussed at a consensus meeting using the nominal group technique. Recommendations were accepted if more than 80% agreement was reached.

## Results

The literature search yielded 523 articles, of which 22 were considered relevant and therefore scored for validity and level of evidence. Eighteen were scored valid and used in the formulation of the recommendations. Seventeen recommendations were suggested in the online survey and discussed during the consensus meeting. Five general recommendations on management, two recommendations for diagnosis, seven for monitoring and eight for treatment were accepted with more than 80% agreement. Topics covered are the following: use of the multidisciplinary team, treatment goals and vaccinations [general recommendations], TNFRSF1A screening, interpretation of R92Q and P46L variants [diagnosis], monitoring frequency and minimal assessments, the use of AIDAI score in clinical studies and the risk of amyloidosis [monitoring], NSAIDS and/or glucocorticoids during attacks, IL-1 blockade, etanercept, anti-TNF monoclonal antibodies, switching between biologicals [treatment].

## Conclusion

The SHARE initiative provides recommendations for diagnosis and treatment for TRAPS and thereby facilitates improvement and uniformity of care throughout Europe.

## Disclosure of interest

N. Ter Haar Grant / Research Support from: SHARE is funded by the European Commission ( project N° 20111202), P. Brogan Grant / Research Support from: Novartis, Roche, Consultant for: Roche, G. Grateau Consultant for: Novartis, J. Anton-Lopez Grant / Research Support from: Abbvie, Novartis, Pfizer, Consultant for: Novartis, Speaker Bureau of: Abbvie, Novartis, Pfizer, Roche, SOBI, K. Barron: None declared., L. Cantarini Grant / Research Support from: Novartis, SOBI, Consultant for: Novartis, SOBI, J. Frenkel Consultant for: Novartis, Speaker Bureau of: SOBI, C. Galeotti Grant / Research Support from: Novartis, V. Hentgen Consultant for: Novartis, M. Hofer: None declared., T. Kallinich Grant / Research Support from: Novartis, Speaker Bureau of: Novartis, SOBI, I. Kone-Paut Grant / Research Support from: Chugai, Novartis, SOBI, Consultant for: Abbvie, Chugai, Novartis, Pfizer, SOBI, Speaker Bureau of: Novartis, Pfizer, J. Kümmerle-Deschner Grant / Research Support from: Novartis, Speaker Bureau of: SOBI, H. Ozdogan: None declared., S. Ozen Consultant for: Novartis, Speaker Bureau of: Biovitrium, A. Simon Consultant for: Novartis, SOBI, Xoma, Y. Uziel Grant / Research Support from: Novartis, Consultant for: Novartis, Speaker Bureau of: Abbvie, Neopharm, Novartis, Roche, C. Wouters: None declared., B. Feldman: None declared., B. Vastert Consultant for: Novartis, N. Wulffraat Grant / Research Support from: Abbvie, GSK, Roche, Consultant for: Genzyme, Novartis, Pfizer, Roche, H. Lachmann: None declared., M. Gattorno Grant / Research Support from: Novartis, Speaker Bureau of: SOBI

